# The views of women and their physicians on decision-making for stress urinary incontinence

**DOI:** 10.1007/s00345-025-05668-4

**Published:** 2025-05-26

**Authors:** Nienke J. E. Osse, Karine Gontijo-Santos Lima, Marian K. Engberts, Hugo W. F. van Eijndhoven, Philippe D. Violette, Rufus Cartwright, Marco H. Blanker, Paul. L. P. Brand

**Affiliations:** 1 Department of Primary- and Long-Term Care, University Medical Center Groningen, Hanzeplein 1, 9713 GZ Groningen, The Netherlands; 2 Department of Obstetrics and Gynaecology, Isala Hospital, Dokter Van Heesweg 2, 8025 AB Zwolle, The Netherlands; 3 Departments of Surgery and Health Research Methods Evidence and Impact, McMaster University, 310 Juliana Dr, Woodstock, ON N4 V 0 A4 Canada; 4 Department of Gynaecology, Chelsea and Westminster NHS Foundation Trust, 369 Fulham Rd., London, SW10 9 NH UK; 5 Department of Medical Education and Faculty Development, Isala Hospital, Dokter Van Heesweg 2, 8025 AB Zwolle, The Netherlands; 6 Wenckebach Institute for Medical Education, University Medical Center Groningen, Hanzeplein 1, 9713 GZ Groningen, The Netherlands

**Keywords:** Patient perceptions, Physician perceptions, Mixed-methods study, Shared decision-making, Stress urinary incontinence, Treatment decision

## Abstract

**Introduction:**

Treatment decisions for stress urinary incontinence (SUI) are preference sensitive, because the disease is non-lethal and there are multiple reasonable treatment options. However, little is known about patients’ and physicians’ preferred decision-making styles for SUI. To aid physicians in their counselling and decision-making in consultations for SUI, we studied patients’ and physicians’ preferred and perceived decision-making in medical specialist consultations for SUI.

**Methods:**

This mixed-methods study combined the validated control preference scale (CPS) and the CPS perception version, and semi-structured, in-depth interviews with both patients and physicians. This study took place in Canada, the United Kingdom and the Netherlands. Sixteen physicians from all three countries and seventeen women from the Netherlands and Canada were interviewed.

**Results:**

All women expressed a preference for being involved in the decision-making process, either by informative or shared decision-making (SDM) in the CPS, because they valued the autonomy to make their own choice regarding treatment for SUI and appreciated receiving information and advice from their doctor. Physicians also preferred an involved patient, but used medical expertise to steer towards their preferred treatment option. Physicians found SDM difficult to understand, expressing different interpretations.

**Conclusions:**

SDM is not a precise concept either for patients or physicians, with multiple interpretations. All patients with SUI want to be involved in the decision-making process, either by informative or by shared decision-making. Physicians both express the desire to involve patients in their decision making, but conversely to steer patients towards the decision that they feel suits them best.

## Introduction

Women’s views on living with stress urinary incontinence (SUI) have been studied extensively [1]. SUI is experienced with feelings of fear, shame and stigmatization, which are felt across different ethnic groups [2]. As SUI is a non-life-threatening disease, there is room for delayed help-seeking behaviour depending on the patient’s willingness to be treated [3]. This is highly influenced by a combination of perceived quality of life, patients’ perceptions of normality of the disease and trust in treatment options [4]. These factors also influence patients’ treatment goals for SUI, which appear to be different from physicians’ treatment goals for SUI. Whilst patients predominantly want to regain their quality of life, physicians put more weight on functional improvement [5]. These differences may affect the way in which the decision for treatment of SUI is being made in medical consultations.

Three decision-making models in medicine are distinguished: paternalistic, informative, and shared decision-making. Paternalistic involves the physician leading decisions; informative allows the patient to decide with provided information; shared decision-making (SDM) balances both, integrating the physician’s expertise with the patient’s preferences, promoting autonomy and informed choices [6, 7]. SDM is associated with better informed, satisfied patients who are more likely to follow medical advice [7]. In SUI, most women prefer SDM, although some are comfortable with their physician deciding for them [8]. When considering treatment options, patients take factors like efficacy, complications, invasiveness and recovery time into account. Some women choose a treatment after eliminating less attractive other options, or prefer to start with the least invasive option [1, 9–11]. However, the limited literature on patients'decision-making preferences gives little insight into the reasons behind their choices, complicating the development of effective patient-centred care strategies.

Physicians also differ in their approach to decision-making [12, 13]. There are large differences between surgeons in their preferred decision-making style and in their personal experiences with operating procedures for SUI [11]. In addition, physicians appear to be unable to adequately describe their decision-making style and overestimate their use of SDM [14]. How physicians view their decision-making process when treating SUI has not been studied to date. Understanding these views is important for improving both the decision-making process and the overall quality of care provided to patients with SUI.

Thus, both patients and physicians differ in their preferred decision-making method for choosing a treatment for SUI, but the reasons for these decision-making differences amongst physicians and patients are unclear. Our research aimed to provide insight in these differences by combining data from three Western countries. To aid physicians in the decision-making process and obtain more insight into the background of patients’ and physicians’ views on decision-making for the treatment of SUI, our mixed-methods study examined treatment decision-making in consultations for SUI, and explored patients’ and physicians’ views on this process.

## Methods

### Study design

This mixed-methods study included women attending either gynaecology or urology outpatient departments for treatment of SUI. From May 2023 to June 2024, participants were included in five secondary care hospitals in Canada, the United Kingdom (UK) and the Netherlands. All participants completed validated questionnaires. In Canada and the Netherlands, patients were interviewed about their experiences with and preferences for the decision-making process. Physicians were interviewed about their views on their decision-making styles in all three countries. We used purposive sampling on age, level of education and severity of symptoms to recruit a diverse patient population. 

### Participants

We included adult women with pure SUI or stress-predominant mixed urinary incontinence, for whom pelvic floor muscle therapy was insufficient. Exclusion criteria were prior incontinence or prolapse surgery, current pregnancy and/or patients’ desire for future pregnancy, and a poor cognitive function, as subjectively assessed by the investigator. The physicians of these women were also included.

### Procedures

After referral to the outpatient clinic, all eligible consecutive patients received information about the study from members of their health care team. All participants provided written informed consent. Participating physicians provided background characteristics once during the study.

Before the outpatient consultation, patients were surveyed to gather clinical and demographic characteristics and establish their preferred decision-making style. We obtained information on marital status, ethnicity, highest level of education, current employment status, smoking status, menopausal status, sexual activity and number of vaginal deliveries and caesarean sections. Height and weight were collected to calculate BMI. We also collected information about patients’ urinary incontinence: the type of urinary loss and duration of symptoms, previous treatments, the Sandvik Incontinence Severity Index (ISI) and the Patient Global Impression of Severity (PGI-S) [15, 16]. Patients’ preferences regarding the decision-making process were assessed with the control preference scale (CPS), a 5-point Likert scale [17].

After the consultation, patient’s perceptions regarding the decision-making process were assessed with a modified CPS (pCPS) [18]. Physicians perceived decision-making style were assessed with the pCPS-Physician Version [18].

Semi-structured, in-depth interviews with patients in the Netherlands were held before and after the consultation, to explore women’s preferences for and perceptions of the decision-making process during the consultation, respectively. For logistical reasons, patients in Canada could only be interviewed after the consultation, while patients in the UK could not be interviewed at all. Physicians were interviewed once, at a time of their convenience.

All interviews were held face-to-face, by phone or by video conferencing, according to participants’ preference. The interviews were audio-recorded, pseudonymized and transcribed verbatim.

Interviews were conducted by the first author, who was trained in qualitative research interviews. The interview guide (see Appendix 1) was developed based on existing literature and discussions within the research team.

We included patients until sufficient information had been obtained to reliably describe patients’ views on decision-making and their preferences and experiences of the consultations, without inconsistencies or major lapses in logic. This applied when no new information became known in two consecutive interviews. Similarly, information sufficiency was reached in the physician interviews.

### Data analysis

Descriptive statistics were used to analyse participants’ background characteristics and their responses to the CPS, pCPS and pCPS-Physician version.

Interview data analysis occurred simultaneously with data collection in an iterative fashion, ensuring that the interview guide was adapted when necessary. The interviews were analysed thematically, using both inductive and deductive coding. Two researchers (NO, KG or a student) read and open coded each transcript independently. After every two to four interviews, the research team discussed discrepancies until agreement was reached about the initial coding list. First, the codes were organised into concepts, which were then developed into themes. These concepts and themes were discussed within the research team until a conceptual level of analysis was reached [19]. Atlas.ti version 24.1 (Scientific Software Development GmbH, Berlin, Germany) and the web-version were used to support the coding and analysis process.

### Ethical approval

Ethical approval was provided in the Netherlands by the Local Assessment Committee of Isala Hospital, reference number 20220809, and of St. Antonius Hospital, reference number R&D/Z23.057. The Health Research Authority and Health and Care Research Wales of the NHS provided ethical approval in the UK with Research Ethics Committee reference number 23/YH/0086. The ethical approval in Ontario, Canada was provided by the Hamilton Health Sciences Integrated Research Ethics Board, reference number 16596, and by the Clinical Research Committee at Woodstock Hospital.

## Results

**Fig. 1:**
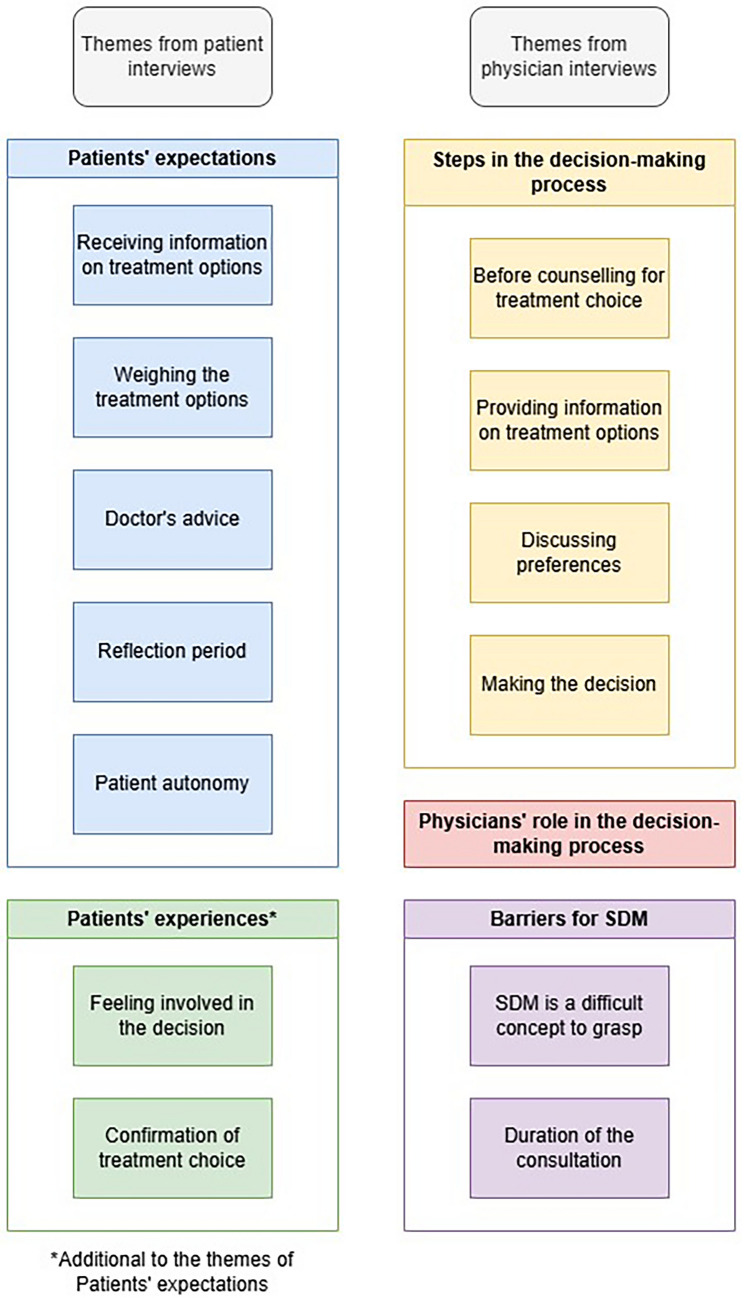
Themes and subthemes identified in the interviews

All representative quotes reflecting codes and themes can be found in Table 3. Each theme consisted of multiple subthemes (Fig. 1).

### Patient interviews: expectations and experiences regarding decision-making style

Data sufficiency was reached after interviewing 17 women, whose characteristics are presented in Table 1 and in Appendix 2. The interviews lasted between 17 and 80 min.

**Table 1:** Patient participant demographics

Demographics	N = 17
Age, mean (SD)	53.1 (12.4)
Ethnicity	
Caucasian descent*, n *(%)	16 (94)
Asian descent*, n *(%)	1 (6)
Level of education	
Secondary education*, n *(%)	8 (47)
Further education*, n *(%)	1 (6)
University education*, n *(%)	8 (47)
Kind of incontinence	
SUI*, n *(%)	10 (59)
MUI*, n *(%)	7 (41)
Duration of complaints (years), median (range)	10 (1–38)
PGIS^a^	
Mild*, n *(%)	4 (24)
Moderate*, n *(%)	6 (35)
Severe,* n *(%)	6 (35)
CPS	
Informative decision,* n *(%)	8 (47)
Shared decision, *n *(%)	9 (53)
pCPS	
Informative decision*, n *(%)	11 (65)
Shared decision*, n *(%)	6 (35)
pCPS-Physician	
Informative decision*, n *(%)	12 (71)
Shared decision,* n *(%)	4 (24)
Paternalistic decision,* n *(%)	1 (6)

*SUI* Stress Urinary Incontinence, *MUI* Mixed Urinary Incontinence, *PGIS* Patient Global Impression of Improvement, *CPS* Control Preference Scale, *pCPS* Control Preference Scale, perception version

^a^One patient is missing

We identified five themes in women’s expectations regarding decision-making prior to their consultation: ‘receiving information on treatment options’, ‘weighing the treatment options’, ‘doctor’s advice’, ‘reflection period’, and ‘patient autonomy’. According to their CPS responses, all women wanted to be involved in the decision-making process: six preferred informative decision-making and eight SDM (Table 1). All women valued the autonomy to make their own choice regarding treatment for their incontinence and appreciated receiving information and advice from their doctor. Some expressed the desire to have some time to reflect, discuss and weigh the options before making a treatment choice.

The 17 interviews about women’s decision-making experiences during the consultation reflected the same five themes, with two additional themes: ‘feeling involved in the decision’ and ‘confirmation of treatment choice’. All women perceived either informative or shared decision-making, according to their pCPS responses. Almost all women were satisfied with the decision-making style they experienced, because it empowered them to make their own choice autonomously (see Q1 in Table 3).

Two women were not completely satisfied, as they felt rushed into making a decision or they did not feel listened to. One of these women had a pessary inserted during the consultation, after which she was interviewed, and she was unsatisfied with the treatment outcome.

#### Receiving information on the treatment options

Before the consultation, the women wanted to be informed about the available treatment options and learn about the benefits and risks of those options for their personal situation. They deemed it important to be able to make an informed choice themselves, based on available relevant information.

Almost all women experienced a thorough explanation of the treatment options. Their doctors drew pictures, talked about the aspects of the options and discussed the numbers with them (Q2). The two women who were unsatisfied stated that they did not understand the explanation completely, and felt that they did not get the opportunity to ask enough questions.

#### Weighing the treatment options

After receiving information about the treatment options from their doctor, the women would prefer to weigh the options, either with their doctor or by themselves (Q3).

Women also experienced that they could weigh the treatment options and their aspects. Some women perceived this as a shared activity with their doctor, while others weighed the options by themselves.

#### Doctor’s advice

The women wanted to hear their doctor’s advice on the preferred course of action, as they regarded their doctor to be an expert in the field. They wanted to hear their doctor’s opinion on the importance of benefits and risks in their specific situation. They also trusted their doctor’s experience to provide a balanced advice, expressing faith in their doctor, believing doctors to have integrity and have their patient’s best interest in mind (Q4). Some women expressed concern that their doctor might not be willing to provide certain treatment options based on the severity of their SUI. These women implicitly assumed that they thought their doctor had the final say in their treatment choice.

Most women reported having asked their doctor for advice when deliberating on a treatment choice. Even when their doctor did not express a preferred treatment option for their specific situation, most women still perceived this as advice from a trustworthy professional (Q5).

#### Reflection period

Some women stated that they would prefer more time to contemplate the options, gather more information, and discuss it with others, after receiving information and advice from their doctor (Q6).

In most consultations, the treatment decision was postponed for a few weeks, either at request from the patient or as suggested by the physician. The women indeed used this reflection period to contemplate, do their own research, or to ask opinions and advice from those around them.

#### Patient autonomy

Before the consultation, participants reported autonomy of their own body as the key reason to want to have the final say about their treatment (Q7).

Most women also experienced autonomy to make a decision regarding their own body, and appreciated having the final say.

#### Feeling involved in decision

Most women felt that their doctor was actively involving them in the decision-making, particularly when the doctor explicitly asked them to express their thoughts of the treatment options (Q8). The two women who were less satisfied with the care they received felt not actively involved in the decision and felt rushed to make a decision (Q9).

#### Confirmation of treatment choice

After making the treatment choice, women appreciated a confirmation from their doctor that they made a good choice (Q10).

### Physician interviews: experiences regarding decision-making style

Sixteen physicians from five hospitals were interviewed. Their characteristics are presented in Table 2, see Appendix 3 for the individual characteristics. The interviews lasted between 23 and 53 min. We identified three themes in physician’s views on decision making for SUI: ‘steps in the decision-making process’, ‘physicians’ role in the decision-making process’, and ‘barriers for SDM’.

**Table 2:** Physician participant demographics

Demographics	N = 16
Age, median (range)	43.5 (29–57)
Gender	
Male, *n *(%)	2 (13)
Female, *n *(%)	14 (88)
Ethnicity	
Caucasian descent*, n *(%)	10 (63)
Asian descent,* n *(%)	4 (25)
African descent,* n *(%)	1 (6)
Middle Eastern descent,* n *(%)	1 (6)

#### Steps in the decision-making process

Physicians discussed four steps in the decision-making process: ‘before counselling for treatment choice’, ‘providing information on treatment options’, ‘discussing preferences’, and ‘making the decision’.

##### Before counselling for treatment choice

Physicians preferred the patient to be well prepared, e.g. through information they had received from their GP or a physical therapist, heard from others or had looked up on the internet. Some hospitals provided information leaflets or decision-aids prior to the consultation. None of the physicians asked their patients directly how they wanted to be involved in the decision-making process, but tended to deduce this implicitly (Q11).

##### Providing information on treatment options

When providing the patient with information on the treatment options, physicians reported factors like efficacy and risk percentages, often made visual in a drawing. Physicians tended to only include those options that they considered to be evidence based and that were available in their hospital. For example, MUS-surgeries were unavailable in the UK at the time of the interviews (Q12). Some physicians always offered all treatment options, while others only mentioned those “realistic treatment” options that they deemed fit for the patient, taking comorbidities and other patient factors into account.

##### Discussing preferences

Physicians actively asked which treatment option patients preferred. However, they hardly ever asked the underlying reason, mostly because patients offered this information themselves, either verbally or non-verbally (Q13). The more physicians preferred a certain treatment option, the more they tried to steer a patient towards that option. This preference could be based on medical grounds or on personal preference for performing a treatment (Q14).

##### Making the decision

One consultation was experienced as paternalistic decision-making, all other consultations were deemed to be either informative or shared decisions (Table 1). The paternalistic decision-making was experienced in the consultation of one of the unsatisfied women.

Almost all physicians stated that they wanted the patient to make an informed decision, especially when they deemed the treatment options to be equivalent. They wanted the patient to feel in control, enhancing their autonomy, and deemed patients to be capable of weighing the options. Physicians sometimes struggled with patients that had already set their mind on a treatment, as they were not always willing to consider other options.

Physicians stated that they only made treatment choices when the patient asked their physician to decide. Some physicians stated that they felt uncomfortable to make a recommendation when a patient would ask them for advice or to make the ultimate decision (Q15). When a physician would recommend a treatment option and the patient agreed, they still felt that the patient made the decision. Physicians felt the need to steer a patient when they felt that the patients’ decision wasn’t sufficiently informed or when they thought a patient couldn’t weigh the options properly. One younger participant speculated that more experienced physicians would steer more, as they are better acquainted with the efficacy-risk trade-off. Overall, the physicians in the UK seemed to be more openly steering in their decision-making style than physicians in the other countries. Some stated that they steered due to patients’ low health literacy (Q16). Physicians in the UK and Canada also mentioned legal aspects to be motivators to steer patients.

In other instances, the patient and the physician collaborated to make the treatment decision. This was based on patient-physician trust and shared responsibility of the decision (Q17). Physicians deemed it important to check the patient’s understanding and to provide advice matching the patients’ situation. One participant thought that he increasingly used shared decision-making in his consultations, as his experience provided more room for patient preferences.

#### Physicians’ views on role in decision-making process

Three different roles were identified in the physician interviews. Physicians described their role in the decision-making process as providing guidance to the patient, as collaborating with the patient, or as helping patients. However, physicians did state that these roles change depending on the needs of patient population and the condition of the patient.

#### Barriers for SDM

Almost all physicians expressed two major barriers in applying SDM: ‘SDM is a difficult concept to grasp’ and the ‘duration of the consultation’.

##### SDM is a difficult concept to grasp

The physicians expressed different concepts of SDM (Q18). For some physicians, SDM was only possible in treatment decisions about survival, while others deemed SUI very suitable for SDM as it is a non-lethal disease. Most physicians thought that SDM was only possible if both the patient and the physician were on the same level of knowledge and physicians needed to check whether the patient understood everything. In contrast, another physician stated that patients only need to be informed enough on their situation to make a decision. Physicians agreed that poor health literacy hampers SDM. Multiple physicians stated that to really apply SDM, physicians needed to provide patients with personalized success and risk percentages, which they deemed not possible at this moment. One physician even stated that SDM is a utopic concept.

##### Duration of consultation

All physicians stated that more time would allow them to talk more about patients’ preferred decision-making style, to discuss treatment preferences, and provide more room for the patient to digest the information. One physician suggested that a reduced administrative burden would leave more time for SDM.

## Discussion

This mixed-methods study examined the current treatment decision-making process in consultations for SUI and explored patients’ and physicians’ views on this process.

In our study, patients wanted to receive information and advice from their doctor, even if their doctor did not state a preference. Patients appreciated a reflection period, wanted to feel involved in the decision, to have autonomy over their body, and receive reassurance from their doctor that they made the right choice. Patients were unsatisfied when they did not feel heard or felt rushed into making the decision. Physicians used multiple ways to make a treatment decision, predominantly wanting the patient to make an informed or shared decision. However, physicians seemed to steer more than they acknowledged. They found SDM a difficult concept to grasp and felt hampered in using SDM, because they lacked personalized success and risk statistics, and felt a shortage of time to properly discuss everything with the patient.

There was considerable overlap between the steps physicians take in the decision-making process and those experienced by patients. Both patients and physicians deemed patient autonomy to be especially important as this is an elective surgery. However, patients wanted the physician to confirm their treatment choice. This is in line with other findings where patients do not only want information, but also their physician’s advice [7, 20].

The difference between informative and SDM was unclear to patients, in view of the almost complete overlap in interview themes between patients from these two groups. Previous SDM research found that patients perceive to be more involved in the decision than an independent observer assesses, raising the question what patients perceive as patient involvement [18]. Although there is limited research on that subject, it is known that patients experience a decision as shared if they agree on the decision with their doctor [21]. Other key parts of SDM patients have mentioned in literature are asking questions, expressing their opinions, considering options and deciding or delegating the decision to their doctor [22]. This would suggest that the outcome of the agreement and the steps in the consultation are more important than the communication process. This is in concordance with our findings that almost all patients experienced to be involved in the decision and were satisfied with it, even though some physicians openly steered their patients. Only those who felt they were insufficiently involved in the decision-making, because they felt rushed or not listened to, were unsatisfied. However, one of these women expressed decisional regret due to an unsatisfactory treatment outcome, which could have altered her view on the consultation [23].

Although physicians wanted their patients to be prepared and ready to engage in the conversation, they failed to inform them that they can be involved in the decision-making, which is the important first step in the SDM process [7]. As stated in previous research, patients who are unprepared for an SDM encounter and don’t get an explicit invitation to participate, don’t really engage in SDM due to the power imbalance that exists between patients and physicians [24].

Most physicians expressed a preference for informative decision-making (i.e., providing all the relevant information and allowing the patient to make the decision), but seem to steer their patient nonetheless. This is in agreement with previous studies, in which physicians were unable to assess their own decision-making style [14]. One of the ways physicians steered patients in our study was by only counselling for treatment options that were available and “realistic” in their professional opinion. This clashes with the moral obligation to present all possible treatment options to a patient, whether they can be provided or not. This obligation is rooted in the principles of informed consent and patient autonomy, which are fundamental in medical ethical guidelines and healthcare law. The physicians in this study stated that they wanted to implement SDM, which is congruent with systematic review findings that physicians in gynaecology have positive attitudes towards SDM [12]. However, also in agreement with earlier studies, our physicians expressed two barriers hampering their application of SDM: a lack of time and difficulty of the concept [12, 25–28].

Overall, patients and physicians across three different Western countries expressed the same themes in their decision-making process. However, differences in healthcare and legal systems seem to affect decision-making styles. In this study, physicians from the UK tended to steer their patients more openly and were more vocal about their preferences if they felt legal pressure or because MUS surgery is currently unavailable in the UK.

The main strength of our study is that it is the first to combine patient and physician perceptions regarding treatment decision-making in SUI. By combining these views, we created an overview of the currently used decision-making process from both patients’ and physicians’ perspectives, which could aid physicians in improving their counselling. Another strength is that the study was conducted in three countries, increasing the findings’ generalisability across the western medical world.

We acknowledge the following limitations. Because most patients in our study were Caucasian, no conclusions can be drawn regarding cultural differences. However, previous research on cultural influences showed that cultural norms factor into the decision-making preferences of Asian-American patients, where patients’ families play a major role in the decision-making process [29]. Another study on decision-making in non-Caucasian breast cancer patients also demonstrated an influence of cultural norms, where some cultures expect paternalistic decision-making based on their expert opinion [30]. Further studies are needed to assess whether this also applies to SUI patients. Another limitation is that interviews with patients from the UK were unavailable due to logistical difficulties. It was also impossible to conduct pre-consultation interviews with patients from Canada. However, some Canadian women still expressed their expectations during the interview, which matched those of the Dutch women. Thus, we are confident that our results reached information sufficiency for both patient populations regarding their expectations and experiences.

## Conclusion

SUI is particularly suitable for SDM because the disease is not life-threatening, treatment decisions are preference sensitive and the number of treatment options is limited. However, SDM seems to be more difficult for patients and physicians than expected. Although all patients definitely want to be involved in the decision-making process, they are unsure what this actually entails and how it can be achieved. In addition, physicians want patients to make an informed-decision or share the responsibility, but steer more than they seem to notice. To improve SDM in the treatment of SUI, physicians need be trained in understanding and applying SDM, and inform their patients about the concept.

## Data Availability

Data is provided within the manuscript. Supplementary information is available upon request.

## Appendix 1: Interview guides

### Interview guide for patients prior to the consultation

#### Goal of the interview and position of the interviewer

The purpose of the interviews is to learn more about how you view potential surgeries as a treatment for your urinary incontinence and how you would like that decision to be made.

I am not part of the medical team. I am conducting research on how patients experience consultations with the gynaecologist, and I would like to discuss this with you. I will be asking you several questions. There are no right or wrong answers here; we are interested in hearing your side of the story: what urinary incontinence means to you and what you hope to achieve from your visit to the gynaecologist. The interview is confidential. You will remain anonymous, and your answers will not be included in your medical record.

#### Topic: treatment aspects

I'd first like to talk with you about the potential surgeries as a treatment for your urinary incontinence. What are your current thoughts on this?

#### Topic: decision-making

Lastly, I would like to talk with you about the decision-making process. You just completed the questionnaire on this topic. Could you elaborate on your answer?

### Interview guide for patients after the consultation

#### Goal of the interview and position of the interviewer

I’m not part of the medical team, I want to learn how patients perceive their care. I would like to hear your side of the story: what your urinary loss means to you and what you hope to achieve with this visit. If any relevant topics will emerge, I could, with your permission, discuss this with your physician. The interview itself is strictly confidential, will be analysed anonymously and will not be part of your medical records.

The goal of the interview is to learn more on the way you view possible treatments and what advantages and disadvantages you consider. In addition, I would like to discuss your urinary loss, how you felt about the visit, and how you perceived the decision making process.

#### Topic: patient satisfaction

You just had the consultation with the physician. How did you experience the visit?

#### Topic: treatment aspects

I would like to discuss your feelings regarding the surgical treatment options. The possible treatment options have been explained to you in detail. What do you think about those?

#### Topic: decision-making

You have just filled in a questionnaire on the decision-making you perceived. Could you please elaborate on your answer again?

### Interview guide for physicians

#### Goal of the interview and position of the interviewer

The goal of this interview is to learn more on how physicians view decision-making styles, particularly for stress urinary incontinence. I would like to talk about your way of making a treatment decision with a patient.

#### Topic: last decision made

Please take the last treatment decision you made with a patient on their stress urinary incontinence in mind. How did that go?

#### Topic: treatment aspects

What aspects do you take into account when making a choice?

#### Topic: role in decision-making

What do you regard as your role in the decision making process?

#### Topic: ideal decision-making

What do you view as the ideal way to make a treatment decision?

## Appendix 2: Demographics of individual patient participants

**Table:**

Study number	Age	Ethnicity	Level of education	Kind of incontinence	Duration of complaints (years)	PGIS	CPS	pCPS	pCPS-Physician	Site
001	43	Caucasian descent	University education	MUI	5	3	3	3	2	Dutch
004	34	Caucasian descent	University education	SUI	5.5	1	2	2	3	Dutch
005	58	Asian descent	Further education	SUI	10	2	2	2	1	Dutch
006	42	Caucasian descent	Secondary education	SUI	13	2	3	2	3	Dutch
008	43	Caucasian descent	University education	SUI	11	2	2	2	1	Dutch
010	47	Caucasian descent	University education	SUI	5	1	2	2	2	Dutch
011	55	Caucasian descent	Secondary education	SUI	25	2	3	3	1	Dutch
039	40	Caucasian descent	Secondary education	MUI	8	2	2	2	2	Dutch
052	79	Caucasian descent	Secondary education	MUI	38	3	3	1	5	Dutch
203	49	Caucasian descent	Secondary education	MUI	1	3	1	2	2	Canadian
205	56	Caucasian descent	University education	SUI	30	2	2	2	1	Canadian
206	66	Caucasian descent	University education	SUI	15	1	3	3	1	Canadian
207	55	Caucasian descent	University education	SUI	10	3	3	3	2	Canadian
208	65	Caucasian descent	Secondary education	MUI	2	3	3	3	1	Canadian
210	40	Caucasian descent	Secondary education	MUI	3	1	3	1	3	Canadian
214	71	Caucasian descent	University education	SUI	11	^a^	2	1	3	Canadian
217	59	Caucasian descent	Secondary education	MUI	1	3	3	3	2	Canadian

*SUI* Stress Urinary Incontinence, *MUI* Mixed Urinary Incontinence, *PGIS* Patient Global Impression of Improvement, *CPS* Control Preference Scale, *pCPS* Control Preference Scale, perception version

^a^Missing

### Explanation of PGIS

**Table:**

PGIS	Severity of incontinence
0	Normal
1	Mild
2	Moderate
3	Severe

### Explanation of CPS and pCPS

**Table:**

CPS		Type of decision-making
1	Patient wants to make the final decision	Informative decision-making
2	Patient wants to make the final decision, after considering doctor's opinion	
3	Patient wants to share responsibility of the final decision with doctor	Shared decision-making
4	Patient wants doctor to make the final decision, after considering patient's opinion	Paternalistic decision-making
5	Patient wants doctor to make the final decision	

pCPS		
1	Patient made the final decision	Informative decision-making
2	Patient made the final decision, after considering doctor's opinion	
3	Patient shared responsibility of the final decision with doctor	Shared decision-making
4	Doctor made the final decision, after considering patient's opinion	Paternalistic decision-making
5	Doctor made the final decision	

## Appendix 3: Demographics of individual physician participants

See Table 3

**Table:**

Study number	Age	Gender	Ethnicity	Site
1	44	Female	Caucasian descent	Dutch
2	56	Male	Caucasian descent	Dutch
3	35	Female	Caucasian descent	Dutch
4	43	Female	Caucasian descent	Dutch
5	35	Female	Caucasian descent	Dutch
6	43	Female	Caucasian descent	Dutch
8	47	Female	Caucasian descent	Dutch
11	46	Male	Caucasian descent	British
12	40	Female	African descent	British
13	45	Female	Asian descent	British
15	36	Female	Asian descent	British
16	57	Female	Caucasian descent	British
21	45	Female	Caucasian descent	Canadian
23	47	Female	Middle Eastern descent	Canadian
24	38	Female	Asian descent	Canadian
25	29	Female	Asian descent	Canadian

**Table 3:** Participants’ quotes

Quotation number	Quotation	Participant, perceived decision-making style
Q1	“Yeah, it was… I have nothing bad to say about this. It went very well, I think. I felt like I was validated, included, educated and participated.”	Patient 207, SDM
Q2	“Yes. He explained everything. He explained our options and the medications and surgeries, and yes. Everything was thorough. I don't think I forgot anything, so I think I'm good.”	Patient 208, SDM
Q3	"Well, she will say what seems the best option to her, I suppose. Or maybe even two options, like we just discussed, I think. […] Of course, ultimately I make the decision myself. I mean, I won't let it be imposed on me. But I do think it's a conversation. And I think I should also take the advice to heart, because otherwise, why am I here? I might as well have just said,'well, you know, just do that.'"	Patient 006, SDM
Q4	“I mean, you know, I really believe that the gynaecologist knows, right, what will work or not work. So I mean, I actually trust that, well, let's be honest, almost blindly."	Patient 006, SDM
Q5	“Well, yes, I think that if, and I'm hoping that if he's a doctor, he wants the best interest for the patient, right, and he's going to be honest about it and he's going to tell me what his opinion is, being honest about it, right? Yeah. So I believe that and […] I accept what you tell me and I'm going to think you mean well with it, right? So I'm going to trust that.”	Patient 203, IDM
Q6	"And when I would have doubts, I would say ‘I'll discuss it at home with my partner, I'll weigh things against each other a bit more, and I'll get back to you’, and then I could also make a decision. But I will definitely take the advice of the doctor into account."	Patient 004, IDM
Q7	"Ultimately, it's of course my body with which something will or will not happen. I had some doubts about the responsibility. Because I do think that the doctor is responsible for advising me well. So that's why I hesitated between those two options. I think I'm very inclined to go along with the doctor. But I have also learned that as a patient, you need to be assertive and say what you want. And I do feel that I am responsible myself for what I ultimately decide to do."	Patient 008, IDM
Q8	“She made the proposal, explaining how it works, and the complications. She drew it out and also asked what I thought of it and what I had in mind. What I had thought of myself.”	Patient 001, SDM
Q9	“I wasn't really asked,"well, do you agree with this, or do you want that? Or…"She just said,"well, we can… you can choose a pessary, and then I'll insert it now,"because she had done an internal examination. So you're sitting in that chair, and yes, immediately actually, they check which size I needed. I didn't really say,"oh yes, that's alright"or… at least, I can't remember her asking directly,"do you agree with this?"or"shall we try that?"or… So she just said,"yes, that's an option."And well, yes. So then she fitted it.”	Patient 011, SDM
Q10	“We actually decided quite quickly. She did explain to me what treatments were available. And I quickly figured out what I really wanted. And she said to me, ‘Well, from what I see now, I think the right choice has been made.’ So, yes…”	Patient 005, IDM
Q11	“So then you try to gauge a little how someone wants to make the decision. I know your questionnaire asked,'Did you ask the patient how they want to make the decision?'Well, I don't think I ever do that. To be honest, that's how I answered it too. But of course, you do that naturally during the conversation.”	Doc 04
Q12	“I think it [MUS] was really good for lots of patients. Yeah, I think it was really good. And there probably is a cohort who really benefited from it and would continue to do so. But I think in the current climate, I wouldn't feel comfortable offering it to people.”	Doc 13
Q13	“And I try to gauge which of the outcomes is important for our patients. I just try and see. Looking at them and whatever. Sometimes some things are very clear to me that they're very important. […] And other people may not react quite as much to me mentioning that particular risk. And so it tells me in a qualitative way, some of the aspects that the patient's taking into consideration more of the information I'm telling them. That seems to be the puzzle. I never know what information that I'm communicating is being prioritized, like is being filtered into part of the decision, you know? […] I try to get them to think about it, but I never, for some reason, don't ask them what is more important to them.”	Doc 21
Q14	“I think anybody who says that they don't [take their personal preference into account] is lying, or deluded. Yes. Obviously. It’s… Certainly when we had midurethral tapes available. I mean trans obturator tapes, in my opinion in the right people, done by the right professional, were a 20 min life changing operation. So yes. I would put people towards that.”	Doc 16
Q15	“I think those (patients without preference) are the hardest because I feel like then I'm, like, responsible for, you know, making their decision and all the potential complications that come with it. I think if the patient has no idea what they want to do, then I always lean towards like going more conservative and I start them with the least invasive options. […] Because I think if a bad outcome comes from like surgery and like I was the one who pushed them to do surgery when it wasn’t necessarily necessary. So I try to avoid convincing someone to do a surgery unless there's like a clear indication of benefit versus risk.”	Doc 25
Q16	“We make a display of good decision-making, trying to give good information, but if you actually interrogate patients about what they understand, they understand nothing at all. Or many understand a very limited amount based on the amount, the amount of input we give, which is why we give them information sheets and decision aids and a lot of opportunity to reflect and multiple appointments before reaching these decisions, but still they don't understand.”	Doc 11
Q17	“I think we shared the decision. But I suppose I guide the patient to what seems appropriate from what she's telling me. And she may not be clear about what those options are, so we come to it together. But I may say that I don't think it's appropriate to do surgery, at this point. Even though that's what she wants. Because of limitations on her recovery, and how I think we may limit the benefit of a procedure, at that time. […] So it might be that there is an intervention we could do then, so it's definitely a shared decision making. But I might have to lead her to what those determinants of that decision are. I think it's again disingenuous to say the patient makes the decision, you have to make that decision together.”	Doc 16
Q18	“We should be sharing the responsibility. But then I feel like I should be a facilitator, so therefore that means that they're making the decision, because that's what the word facilitate means. But then if 90% of the time they make the choice that I think that they should make, then what does that mean? Does that mean that I've facilitated them to choose what I think they should choose? […] I think I'm going to go for we are doing it together, and it just turns out that because I have done the job so well, that most of the time, because I've explained it so well, then maybe that's why they're choosing what I thought was in their best interests.”	Doc 12

## Abbreviations

CPS: Control preference scale
ISI: Incontinence severity index
PGI-S: Patient global impression of severity
pCPS: Control preference scale modified to perceptions
SDM: Shared decision-making
SUI: Stress urinary incontinence
UK: United Kingdom
